# Genetic Profile of Linezolid-Resistant *M. tuberculosis* Clinical Strains from Moscow

**DOI:** 10.3390/antibiotics10101243

**Published:** 2021-10-13

**Authors:** Anastasia Ushtanit, Yulia Mikhailova, Alexandra Lyubimova, Marina Makarova, Svetlana Safonova, Alexey Filippov, Sergey Borisov, Danila Zimenkov

**Affiliations:** 1 Center for Precision Genome Editing and Genetic Technologies for Biomedicine, Engelhardt Institute of Molecular Biology, Russian Academy of Sciences, 119991 Moscow, Russia; ushtanit@mail.ru (A.U.); eimb@yandex.ru (A.L.); 2 The Moscow Research and Clinical Center for Tuberculosis Control, Moscow Government Health Department, 107014 Moscow, Russia; juliaisaeva81@rambler.ru (Y.M.); makarova75@yandex.ru (M.M.); safonova.s.g@inbox.ru (S.S.); alex.phil.2010@yandex.ru (A.F.); sebarsik@gmail.com (S.B.)

**Keywords:** tuberculosis, drug-resistance, linezolid

## Abstract

Background: Linezolid, bedaquiline, and newer fluoroquinolones are currently placed as priority Group A drugs for the treatment of drug-resistant tuberculosis. The number of reported linezolid-resistant clinical strains is still low, and the correlation of molecular determinants with phenotype is not perfect. Methods: We determined the linezolid MICs for clinical isolates from the Moscow region and identified mutations in *rplC* and *rrl* genes. Results: All 16 linezolid-resistant isolates had previously reported mutations in the *rplC* or *rrl* loci, and 13 of them bore a RplC C154R substitution. Detection of this substitution in a heteroresistant state was not successful, probably, due to the more stable DNA secondary structure of the mutated fragment, which precludes its amplification in mixes with the wild-type DNA. Strains with an *rplC* mutation had higher linezolid MIC compared to isolates with *rrl* mutations. Conclusions: Linezolid resistance mostly emerged during treatment with the latest regimen. Three primary cases with linezolid resistance question the possible transmission of totally drug-resistant tuberculosis in the Moscow region, which demands further investigation.

## 1. Introduction

Multidrug-resistant (MDR) tuberculosis is a global public health threat, and three countries—India, China, and Russia—account for almost half of all cases [1]. Tuberculosis is intrinsically resistant to many antibacterial drugs, such as beta-lactams, tetracyclines, and macrolides, due to its thick and waxy cell envelope and the presence of drug-modifying enzymes [2]. Resistance to the most effective first-line drugs (rifampicin and isoniazid) defines multidrug-resistant (MDR) tuberculosis, and additional resistance to second-line core drugs (injection drugs and fluoroquinolones) defined the extensively drug-resistant (XDR) tuberculosis up to 2020. The treatment of MDR and XDR tuberculosis is a major challenge, due to the limited number of effective drugs [3]. Moreover, medication should include at least five effective antituberculosis drugs [4]. The recently developed drugs, bedaquiline and delamanid, and the repurposed linezolid, clofazimine, and carbapenems, provide novel opportunities for fighting tuberculosis [5]. Linezolid, bedaquiline, and the newer fluoroquinolones are currently placed as priority Group A drugs, endorsed by the WHO for resistant tuberculosis treatment [6]. Consequently, a new definition of XDR tuberculosis was introduced owing to the data on the low efficiency and serious side effects of second-line injection drugs [7].

Linezolid and bedaquiline were introduced as cornerstone components of novel therapy in 2014 in Russia and resulted in a noticeable improvement in outcomes [8]. Resistance of clinical isolates to linezolid is still rare, compared to the growing number of reports about bedaquline-resistant clinical strains [9].

The main mechanism of linezolid resistance in *M. tuberculosis* is the C154R substitution in ribosomal L3 protein, encoded by *rplC* gene [10]. Mutations in 23S rRNA gene *rrl* are found less frequently; however, they are located at different sites along the gene, which are responsible for forming the peptidyl-transferase center of the ribosome [11]. Other plasmid- or transposone-borne mechanisms of linezolid-modifying enzymes, which are found in *Enterococcus*, *Staphylococcus*, and *Streptococcus* [12], are absent in *M. tuberculosis*, due to the absence of horizontal transfer of genes. However, recent findings of resistant isolates with no mutations in known loci, prompt questions about the existence of other mechanisms of resistance [9,13].

Here we analyzed the phenotypic and genetic properties of clinical linezolid-resistant *M. tuberculosis* strains, isolated from patients in Moscow region between 2017 and 2020.

## 2. Results

Phenotypic linezolid resistance determination has been systematically performed at Moscow Research and Clinical Center for Tuberculosis Control protocols since December 2016. In total, cultures from 322 patients, who were scheduled to be treated with a bedaquiline- and linezolid-containing regimen, were analyzed. Forty-eight of the patients had previously been treated with linezolid. In total, 20 TB cases with acquired or primary linezolid resistance were identified between January 2017 and November 2020. Four cultures could not be regrown for MIC determination and genotyping and were omitted from the study. All isolates were XDR, according to the novel WHO definition: resistance to first-line drugs plus fluoroquinolones and linezolid [7].

### 2.1. Detection of rplC t460c Mutation in Heteroresistant State

Alteration of the ribosome is the main mechanism of resistance to linezolid in *M. tuberculosis*. According to previous reports we performed sequencing of *rrl* and *rplC* fragments. However, initially we failed to detect any mutations in the two resistant isolates. One of these (#12) was subjected to whole genome sequencing using the Illumina MiniSeq platform. The average depth of coverage was 454× after mapping to the reference H37Rv genome. About 20% of reads (93 of 470 total) in the *rplC* 154 codon region displayed the well-described mutation t460c, leading to C154R substitution (Figure S1). The other 80% corresponded to the wild-type sequence.

**Figure 1:**
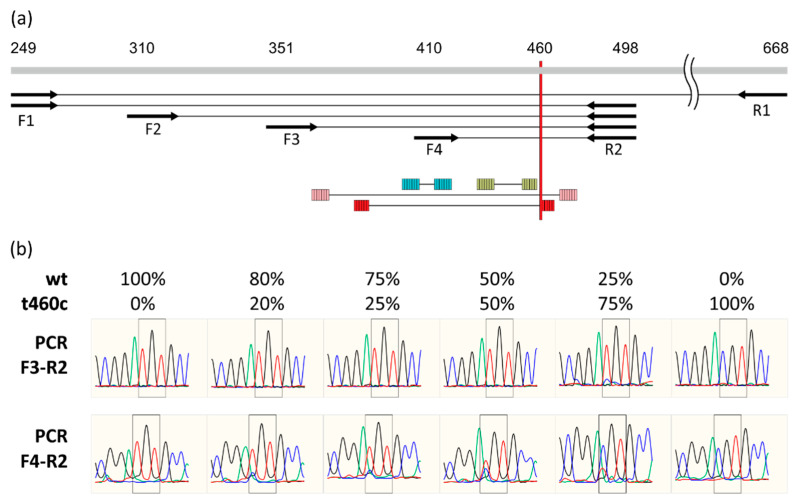
Amplification and sequencing of the *rplC* locus. (**a**) Positions of primer binding sites and DNA secondary structure, the additional stem forming due to t460c mutation is shown with red. (**b**) Sequencing chromatograms with artificial mixes of wild-type and mutant genomic DNA in various ratios.

We proposed that the amplification efficiency for DNA with t460c mutation is lower than for wild-type DNA, so predominately only the latter is amplified during PCR and Sanger sequencing of the mixed samples. We designed a set of PCR primers located upstream and downstream of the 154 codon (Figure 1a). The model mixes that contain various proportions of a mixture of wild-type and mutant genomic DNAs were used as amplification targets and for further sequencing. Indeed, using the first set of primers (F1-R1), even a 75% mutant DNA prevalence resulted with only wild-type peaks on the chromatogram. Substitution of the reverse primer to R2 did not improve the detection sensitivity. The same was true when F2 or F3 were used instead of F1 as an upstream primer. However, the amplification with primers F4 and R2, which resulted in the shortest fragment, allowed for the reliable detection of mixes, starting from 20% of mutated DNA prevalence (Figure 1b).

Based on the drastic difference in amplification with the two closely located primer binding sites, F3 and F4, we proposed that the secondary DNA structure affects the amplification efficiency. We compared the predictions of secondary structure for wild-type and mutated sequences using the mfold algorithm. Indeed, a t460c substitution lead to the formation of additional 6 bp stem (5′-ggcacg-…-cgtgcc-3′, g460c underlined), compared to the wild-type sequence (Figure 1a). Thus, mutated DNA could form a more stable branched hairpin structure.

### 2.2. Genetic and Phenotypic Properties of Clinical Isolates

We performed 24-locus mycobacterial interspersed repetitive unit–variable-number tandem repeat (MIRU–VNTR) typing and targeted the Sanger sequencing of *rplC* and *rrl* loci, associated with resistance to linezolid.

The MIRU–VNTR genotyping results were compared to the MIRU–VNTRplus database [14]. Genotypes of the five isolates had perfect matches in the database. Most of the others had 1–2 repeats difference in the 1–2 loci (Table 1), and the isolate genotypes were determined as a genotype from the best matching isolate from the MIRU-VNTRplus database. One isolate (#10) differed from the best matching in as much as seven loci. Searching for the closest isolate type in the published study by Merker [15] resulted in the identification of the clinical strain OB107, which differs only in three loci (2, 7, 3, 3, 3, 5, 2, 4, 4, 4, 2, 3, 6, 4, 7, 2, 5, 3, 2, 1, 3, 4, 2, 3; differences underlined). This strain was isolated in Korea and belongs to the ancestral Beijing BL7 clonal complex, type 9515-32.

Most of the isolates belonged to the Beijing lineage and the predominant types were 94-32 (*n* = 6 isolates) and 100-32 (*n* = 4 isolates). The latter genotype corresponded to the successful Russian clone, B0/W148 [16,17]. Three isolates belonged to Lineage 4: two different LAM genotypes and one Ural 163-15 [18]. One isolate (#15) exhibited a heterogeneous MIRU-VNTR allele profile, reflecting mixed infection with different strains. Moreover, it bore a *rplC* mutation in the heteroresistant state.

The main determinant of linezolid resistance was a C154R substitution in RplC; found in 13 of 16 clinical isolates (Table 1). In two cases, this substitution was found in mixed state, with the wild-type sequence: #12 and #15. Both samples had a linezolid MIC of 2 mkg/mL, which is lower than the mode of MIC distribution for isolates with this substitution (4 mkg/mL, *n* = 7 strains).

Three isolates bore mutations in a 23S rRNA gene previously found in clinical strains. A strain with a g2270t mutation had an MIC of 1 mkg/mL, which is below the breakpoint. However, it was found to be resistant by MGIT 960 testing at a critical concentration of 1 mkg/mL. Interestingly, one isolate bore a double mutation in the 23S rRNA gene at the 2810 and 2814 positions (#5).

### 2.3. Clinical Aspects of Linezolid Acquisition

Four cases from a total of 16 were lost before follow-up. One of them was lost due to migration, and no information about the patient and TB clinics is available.

Three cases were primary cases, three were relapses, one was re-enrolled in treatment after loss before follow-up during the most recent course. The other nine cases were switched to a linezolid-containing regimen after the previously ineffective treatment (Table 2).

Thirteen patients were male and three female, aged from 27 to 63 years, with an average age of 40.1 and median of 38.8.

Among the clinical forms of tuberculosis, fibrous-cavernous tuberculosis prevailed in eight patients; one was diagnosed with cirrhotic tuberculosis with large cavities and bacterial excretion; in three the process was interpreted as disseminated tuberculosis with disintegration, with a cavity size of more than 3 cm; infiltrative was established in two patients; and one had caseous pneumonia. In one case (#11) TB meningitis was diagnosed, without pulmonary involvement. Due to sputum negativity, a culture was obtained from cerebrospinal fluid by lumbar puncture in this case.

Two patients with newly diagnosed tuberculosis and another with a relapse of tuberculosis (judging by the differences in the spectrum of drug resistance of the first episode of tuberculosis and relapse, this relapse can be called a recurrent disease) since the detection of resistance to linezolid did not receive a single dose of the drug, therefore they were infected with a strain already resistant to linezolid. In other patients, from six months to three years passed from the first dose of linezolid to the detection of MBT resistance. Moreover, in five of them, the identification of drug resistance to linezolid was preceded by the isolation of an MBT culture susceptible to this drug. In three patients from whom initially resistant strains were isolated, resistance to this drug was also determined when the test for sensitivity to linezolid was repeated, from one to three times (data not shown).

The majority of cases (13 of 16) with linezolid resistance were patients with long-term chronic destructive pulmonary tuberculosis, poor adherence to treatment with long-term (from six months to two or more years) inclusion of linezolid in the regimen, and a history of emergence of drug resistance to other drugs.

## 3. Discussion

In our study, we analyzed linezolid-resistant strains isolated from patients attending the Moscow Research and Clinical Center for Tuberculosis Control. From 2017 to 2020, 16 strains were identified, confirming the previous observations about low rate of resistance development [9].

MIRU-VNTR genotyping confirmed the predominant spread of the Beijing lineage in Moscow region. The majority of isolates (*n* = 10) belonged to the Central Asian/Russian Beijing 94-32 cluster subtype and B0/W148 (type 100-32) clone. Beijing clusters 94-32 and 100-32 were the largest clusters in Russia [19], Central Asia [20], Portugal, and Guinea-Bissau [21]. The differences found in the MIRU profiles of isolates in comparison with MIRU-VNTRplus database question the reliability of sublineage identification in several cases. In one isolate, we had to include additional data from the publication of Merker et al. [15], due to the differences found in 7 of 24 loci, with the closest match from MIRU-VNTRplus.

All linezolid-resistant isolates in our study had previously reported mutations in either *rplC* or *rrl* genes. Recent studies about clinical linezolid resistance raise questions about the existence of mechanisms other than mutations in *rplC*, *rplD*, and *rrl*. In an article by Zheng et al., two of four resistant strains bore *rplC*, while no mutations were found in the other two isolates [22]. Similar results were obtained in other studies: in a study by Du at al. nine of 19 clinical isolates had no mutations [9]; while in in a study by Zong et al. it was six isolates from thirteen [23]. On the other hand, in a study by Wasserman et al. mutations were found in all 16 resistant isolates [24].

One technical point could be the cause of this discrepancy; we proposed that the identification of *rplC* mutations in a heteroresistant state was unsuccessful due to the secondary structure of this locus with a t460c mutation, which lowers the PCR amplification efficiency and Sanger sequencing.

Initially we failed to detect any mutations in two strains by Sanger sequencing. For identification of unknown resistance determinants, next-generation sequencing was performed. However, instead of novel determinants, the most prevalent mutation, *rplC* t460c, was identified in a heteroresistant (mixed with wild-type sequence) state. The same approach and same findings were described in a paper by Zhang et al. [25]. The redesigning of the PCR primers to amplify a shorter fragment allowed obtaining mixed chromatograms and the proper identification of this mutation in a heteroresistant state. This amplification artifact was confirmed by the amplification and sequencing of mixed genomic DNAs from wild-type and mutant strains in different ratios. We found that upstream, and not downstream, DNA affects the detection limit of mutated DNA. Even a 75% content of mutated DNA cannot be identified using amplification of longer fragments, while shorter fragment sequencing resulted in the identification of 20% content mixes with wild-type DNA. Using the mfold algorithm a branched 2-headed DNA hairpin could be identified in this region, and the t460c mutation forms an additional 6-bp long stabilizing stem. Since large fragments are usually used for Sanger sequencing, the reported failures in the detection of mutations in linezolid resistant strains could be due to this phenomenon.

Heteroresistance and mixed infections are not rare in TB high burden regions [26,27], with an estimated prevalence up to several tens of a percent [28]. Mixed infections are more common among patients with immunosupression and a chronic condition [28]. Indeed, an sample with a mix of two different genotypes, was isolated from an HIV-positive patient not previously treated with linezolid. A second sample with an ambiguous sequence of *rplC* locus was isolated from a previously treated patient, also as the most studied isolates. Such heteroresistance, when both wild-type and resistance-associated mutation are detected simultaneously, is often found during treatment and reflects a transition state of the pathogen, leading to the emergence of resistance [29].

Finally, 13 of 16 total resistant isolates bore RplC C154R substitution in our study. Omitting the heteroresistant strains, this substitution was associated with higher linezolid MIC compared to mutations, identified in the 23S rRNA gene. Similar differences in MIC levels were noticed in previous studies [9,24,25]. In addition, it could be shown, that a *rrl* g2270t mutation led to lower MIC (1 mkg/mL), compared to MIC of isolates with other *rrl* mutations (4 mkg/mL). However, the number of strains was too low to draw statistically significant conclusions, and too few linezolid-resistant clinical isolates have been described to date.

Linezolid resistance was associated with poor outcomes, independently of the particular mutation or strain genotype. In conclusion, the three observed primary TB cases with linezolid resistance prompt questions about the possible transmission of totally-drug resistant tuberculosis in the Moscow region, which demands further investigation.

## 4. Materials and Methods

### 4.1. Mycobacterium Tuberculosis Strains

The *M. tuberculosis* strains were obtained from clinical specimens collected from TB patients at the Moscow Research and Clinical Center for Tuberculosis Control. Drug-susceptibility testing for rifampicin, isoniazid, streptomycin, ethambutol, pyrazinamide, ofloxacin, moxifloxacin, kanamycin, capreomycin and amikacin, PAS, and ethionamide was performed using a Bactec MGIT 960, as described previously [30,31]. Critical concentrations for kanamycin, amikacin, and capreomycin were 2.5 μg/mL, 1.0 μg/mL, and 2.5 μg/mL, respectively. Linezolid (Sigma-Aldrich Co., St. Louis, MO, USA) resistance was determined using a MGIT 960 (critical concentration of 1mg/L) [32]. Linezolid MIC was determined using a Middlebrook 7H9 broth microdilution method. Briefly, two-fold serial dilutions of linezolid (Sigma-Aldrich, St. Louis, MO, USA) were made in 7H9 medium supplemented with OADC in 96-well U-bottomed plates. Cell suspensions were adjusted to a 0.5 McFarland standard and diluted 1:100. Plates were incubated for 10 days. Additional incubation for 4–10 days was performed in the cases when insufficient growth was detected in the control wells.

The study was approved by the Ethics Committee of the Moscow Government Health Department. The Ethics Committee waived the need for patient consent because the study did not include any personal identifiers or clinical data and the samples were analyzed anonymously.

### 4.2. DNA Isolation and Sequencing

DNA isolation and sequencing of the *rplC* and *rrl* fragments were performed as previously described [33]. Fragments for sequencing the *rplC* gene were obtained by PCR with the following primers: F1: 5′-gctgcggctggacgactc-3; F2: 5′-gagatcttcgccgatggcag-3′; F3: 5′-ctccaagggcaaaggtttcg-3′; F4: 5′-ccagtcacggtgcccag-3′; R1: 5′-ctcttgcgcagccatcacttc-3′ R2: 5′-catccgggtgcccttgaac-3′.

### 4.3. MIRU-VNTR Typing

Twenty-four loci MIRU-VNTR typing was performed according to [34]. Profiles in the article are given in the following order: MIRU04(ETRD-1), MIRU26, MIRU40, MIRU10, MIRU16, MIRU31(ETRE), Mtub04, ETRC, ETR-A, Mtub30, Mtub39, Qub4156, Qub11b, Mtub21, Qub26, MIRU02, MIRU23, MIRU39, MIRU20, MIRU24, MIRU27 (Qub5), Mtub29, ETRB, Mtub34. The MIRU profiles were compared online to MIRU-VNTR *plus* (http://www.miru-vntrplus.org/ (accessed on 25 August 2021) [14].

### 4.4. Whole-Genome Sequencing and Bionformatic Analysis

Strains for whole-genome sequencing was recultured on Lowenstein-Jensen media for approximately 4 weeks at 37 °C and then heat-inactivated. Genomic DNA was extracted using the Gentra Puregene Yeast/Bact. Kit (cat no. 158567, QIAGEN, Venlo, The Netherlands).

DNA libraries were prepared using an Illumina DNA Prep kit and sequencing was performed using a MiniSeq High Output Kit (300 Cycles) on the MiniSeq platform (Illumina, San Diego, CA, USA).

The sequencing data in the FastQ format were analyzed using the Galaxy web platform at the public server usegalaxy.org [35]. The reads were trimmed using the Trimmomatic tool [36], mapped to the *M. tuberculosis* reference genome (GenBank accession NC_000962.3) [37] with BWA-MEM [38], and refined using BamLeftAlign [39]. Variant calling was performed using FreeBayes [39] and filtered with the VCFlib toolkit. Variant annotation was performed using SnpEff [40]. Further bioinformatic analysis of the obtained SNPs was performed with custom Python scripts. Raw sequence reads were submitted to the NCBI SRA server (accession SRR16168839).

DNA secondary structure prediction were performed using the mfold server [41].

## 5. Conclusions

Linezolid-resistant tuberculosis emerged during treatment with novel regimens. The major mecahnism of resistance C154R substitution in ribosomal protein RplC. Mutations in 23S rRNA gene are more diverse, and are associated with lower MICs. Three primary cases with linezolid resistance question the possible transmission of XDR or even, totally drug-resistant, tuberculosis in the Moscow region, which demands further investigation.

## Figures and Tables

**Table 1:** Phenotypic and genetic characterization of the isolates.

ID	MIRU-VNTR ^1^	Genotype	Beijing Clonal Complex	LZR Phenotype			
				MGIT	MIC	*rrl*	*rplC* ^2^
#1	255432322122236152213423	LAM 121-52	-	R	8	wt	t460c
#2	252422322122236162213423	LAM 121-51	-	R	8	wt	t460c
#3	253335444432658253213423	Beijing 94-32	CC1	R	1	g2270t	wt
#4	253336444442658253213423	Beijing 94-32	CC1	R	4	wt	t460c
#5	273334444432656253213423	Beijing 98-32	CC3	R	4	a2810c; g2814t	wt
#6	274335344432657253213423	Beijing 100-32	CC2	R	4	wt	t460c
#7	213722354433236252213423	URAL 163-15	-	R	4	wt	t460c
#8	273335444432657253213423	Beijing 100-32	CC2	R	4	wt	t460c
#9	253335444432658253213423	Beijing 94-32	CC1	R	4	wt	t460c
#10	274334244423647252213423	Beijing 9515-32	BL7	R	4	wt	t460c
#11	253335443432658253213423	Beijing 94-32	CC1	R	16	wt	t460c
#12	273335444432657253213423	Beijing 100-32	CC2	R	2	wt	(t460c)
#13	253334444232658253213423	Beijing 94-32	CC1	R	2	wt	t460c
#14	273345444432657253213423	Beijing 100-32	CC2	R	4	g2814t	wt
#15	2(7/6)3(3/1)4544(3/4)432 (5/6)(4/5)8253213423	mixed	-	R	2	wt	(t460c)
#16	253346444442658253213423	Beijing 94-32	CC1	R	4	wt	t460c

^1^ The differences from the MIRU-VNTR *plus* database are underlined. ^2^ The mutations in parenthesis are present in the heteroresistant state.

**Table 2:** Resistance profiles of the isolates and clinical characteristics of the patients.

ID	Genotype	Drug resistance Profile ^1^		Case	TB Form ^2^	HIV	Days Form Treatment Start	Outcome
		**H.R.Z.E.S**	**Fq.Sl.Ps.Et.Cs**					
#1	LAM 121-52	R.R.R.R.R	R.R.R.R.S	Previous ineffective course	Cirrhotic	-	769	Failure
#2	LAM 121-51	R.R.S.R.S	R.R.R.R.S	Chronic	Fibro-cavernous	-	392	Lost to follow up
#3	Beijing 94-32	R.R.R.R.S	R.S.S.S.S	Chronic	Fibro-cavernous	-	267	Failure
#4	Beijing 94-32	R.R.R.R.R	R.R.R.R.S	Primary	Infiltrative	-	398	Death
#5	Beijing 98-32	R.R.R.R.R	R.R.R.R.S	Chronic	Fibro-cavernous	-	832	Death
#6	Beijing 100-32	R.R.R.R.R	R.R.R.R.S	Relapse	Caseous pneumonia	-	0	Death
#7	URAL 163-15	R.R.R.R.R	R.R.R.R.S	Chronic	Fibro-cavernous	-	206	Lost to follow up
#8	Beijing 100-32	R.R.R.R.R	R.R.R.R.S	Relapse	Disseminated	-	1052	Death
#9	Beijing 94-32	R.R.S.R.R	R.S.R.S.S	Chronic	Fibro-cavernous	-	386	Failure
#10	Beijing 100-32	?.?.?.?.?	?.?.?.?.?	ND	ND	ND	ND	Lost to follow up
#11	Beijing 94-32	R.R.R.S.R	R.S.S.R.S	Primary	TB meningitis	-	0	Death
#12	Beijing 100-32	R.R.R.S.R	R.R.R.R.S	Chronic	Fibro-cavernous	+	1072	Failure
#13	Beijing 94-32	R.R.S.S.R	R.R.R.R.R	Chronic	Fibro-cavernous	-	984	Failure
#14	Beijing 100-32	R.R.S.R.R	R.R.R.R.S	Relapse	Infiltrative	-	876	Lost to follow up
#15	mixed	R.R.R.R.R	R.R.S.R.S	Primary	Disseminated	+	0	Death
#16	Beijing 94-32	R.R.R.S.R	R.R.R.R.S	Relapse	Disseminated	+	330	Death

^1^—resistance to streptomycin (S), pyrazinamide (Z), rifampicine (R), isoniazid (H), ethambutol (E), fluoroquinolones (Fq), second-line injection drugs (Sl), para-aminosalicylic acid (Ps), and ethionamide (Et) was detected using Bactec MGIT 960, and to cycloserine (Cs) using LJ media. ^2^—according to Russian classification of diseases.

## Data Availability

The data presented in this study are openly available from the NCBI SRA archive under the accession SRR16168839.

## Supplementary Materials

The following are available online at https://www.mdpi.com/article/10.3390/antibiotics10101243/s1, Figure S1: Whole-genome sequencing results of #12 strain.
