# Supplementary figures and images for: Genetic Profile of Linezolid-Resistant M. tuberculosis Clinical Strains from Moscow
Source: Antibiotics (Basel). 2021 Oct 13;10(10):1243. doi: 10.3390/antibiotics10101243 (PMC8532644; doi:10.3390/antibiotics10101243)

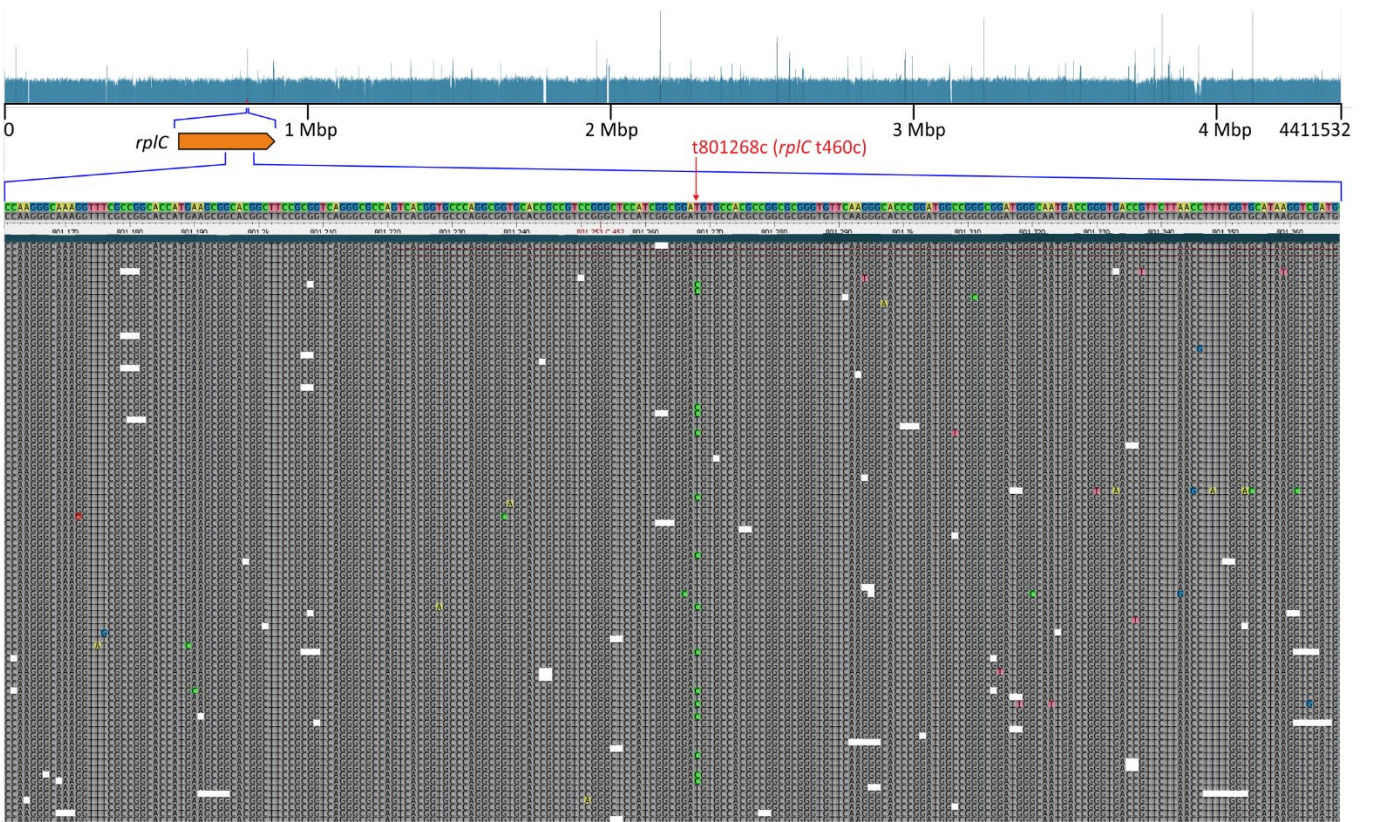

Supplement: Supplementary file 1 [file antibiotics-10-01243-s001.zip › antibiotics-1395477-supplementary.pdf]
